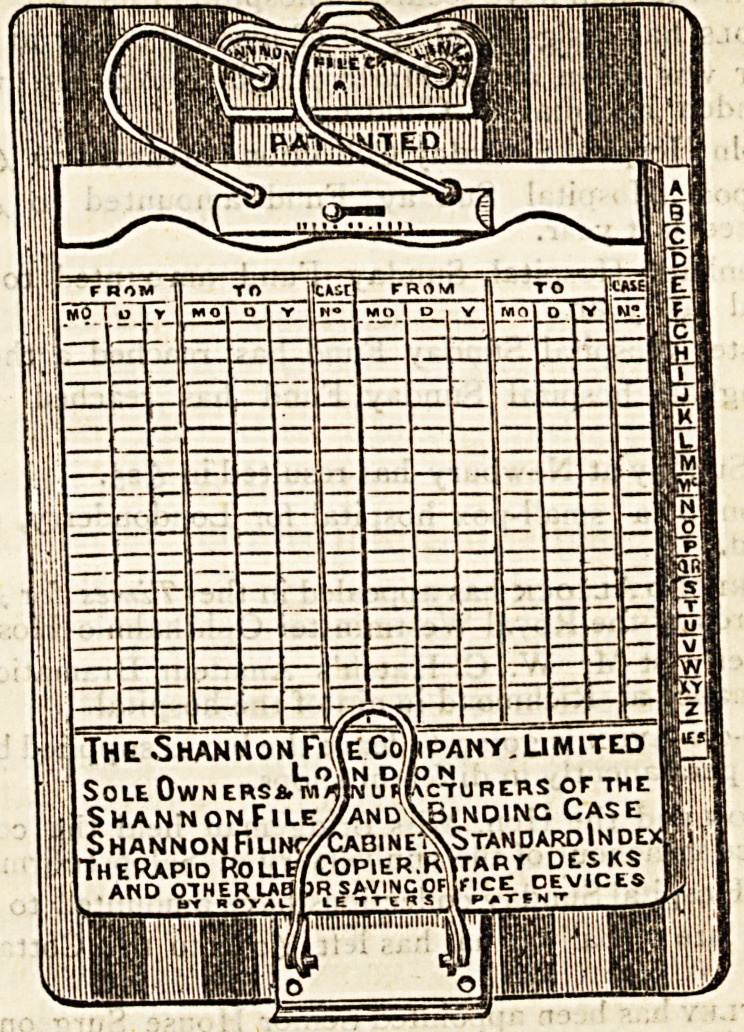# New Remedies and Appliances

**Published:** 1888-11-17

**Authors:** 


### NEW REMEDIES AND APPLIANCES.

THE Shannon File, of which we here give an illustration, was introduced into this country a few years ago with great success. Many other methods of filing letters have been suggested, and great competition has resulted. Competition has led to great improvements in the method of production, and on his return from a recent visit to the Shannon File Company's works at Rochester, New York, Mr. F. W. Schafier announces that his company, while improving the quality of the goods, will shortly be able to supply the public with letter and bill files, cabinets, &c., at prices which will incontestably convince the mercantile public that they are the *cheapest* in the market, while at the same time being second to none in quality. For secretaries of institutions, as well as for professional men and merchants' offices, they will be found to be all that the manufacturers claim for them. They have been in use for over a year in THE HOSPITAL office, and the esteem in which they are held may be gathered from the fact that the occasion for writing this notice arose from the necessity of ordering a fresh supply.

## Figures and Tables

**Figure f1:**